# Characterizing key misconceptions of equity in health financing for universal health coverage

**DOI:** 10.1093/heapol/czac041

**Published:** 2022-06-09

**Authors:** John E Ataguba, Grace A Kabaniha

**Affiliations:** Health Economics Laboratory, Department of Community Health Sciences, Max Rady College of Medicine, Rady Faculty of Health Sciences, 771 McDermot Avenue, University of Manitoba, Winnipeg, MB R3E 0T6, Canada; Health Economics Unit, School of Public Health and Family Medicine, Health Sciences Faculty, University of Cape Town, Anzio Road, Observatory 7925, South Africa; Partnership for Economic Policy, Duduville Campus, Kasarani, Nairobi 00621, Kenya; Health Systems Strengthening Division, World Health Organization Country Office-India, RK Khanna Tennis Stadium, Africa Avenue, New Delhi 110029, India

**Keywords:** Equity in health financing, universal health coverage, income distribution

## Abstract

Fairness or equity in health financing is critical to ensuring universal health coverage (UHC). While equity in health financing is generally about financing health services according to ability-to-pay, misconceptions exist among policymakers, decision-makers and some researchers about what constitutes financing health services according to ability-to-pay or an equitably financed health system. This commentary characterizes three misconceptions of equitable health financing—(1) the misconception of fair contribution, (2) the pro-poor misconception and (3) the misconception of cross-subsidization. The paper also uses these misconceptions to clearly illustrate what constitutes equity in health financing, highlighting the importance of income distribution. The misconceptions come from the authors’ extensive engagements with policymakers and practitioners, especially in Africa. A clear understanding of equity in health financing provides an avenue to significant progress towards UHC and improving a country’s income distribution.

Key messages

- Assessing equity in health financing should consider how income is distributed and not just about looking solely at household healthcare contributions.
- It is not always equitable and fair even when the share of total healthcare contributions by poorer population groups is lesser than the share contributed by more affluent population groups.
- Equity in health financing is relevant on its own even when monetized benefits from using health services are fairly distributed according to need.

## Introduction

Universal health coverage (UHC) is about ensuring that everyone has access to needed health services of sufficient quality to be effective, without any financial hardships to potential service users (World Health Organization, 2010). Fairness or equity in health financing is critical to ensuring UHC. Borrowing from Braveman and Gruskin (2003), although there are nuances and differences in conceptions, we consider equity and fairness as synonyms in this paper. Health services are financed through various mechanisms in many countries, including direct out-of-pocket spending, taxes and health insurance. It is critical to understand, within countries, what it means to finance health services via each mechanism and the entire health financing system comprising all financing mechanisms. While equity in health financing for each financing mechanism and the entire health financing system may be understood as financing health services according to ability-to-pay, misconceptions exist about what constitutes financing health services according to ability-to-pay or an equitably financed health system, perhaps because ‘equity’ or ‘fairness’ is normative (Mooney, 1983). The misconceptions make it difficult for policymakers, decision-makers and some researchers to fully understand equity in health financing and assess progress towards equity in health financing for UHC (Ataguba, 2016). This paper provides an overview of three misconceptions about equitable health financing and illustrates what constitutes equity in health financing. These misconceptions come from our extensive engagements with policymakers and practitioners, especially in Africa.

## Misconceiving equity in health financing

### Misconception 1 (fair contribution)

It is always equitable and fair when the ‘total contributions’ made per head by poorer population groups towards financing health services are lesser than those by wealthier groups.

This misconception of ‘fair contribution’ is illustrated in Table 1 (column d) using a hypothetical example. In that column, the poorest 20% of the population contributes <$100 per head compared to the wealthiest 20%, contributing over $7000 per head per annum. Unfortunately, while a gradient that ‘favours’ the poor exists, it does not reflect the gradient implied by the income distribution in column c, summarized in column b (Table 1). In this scenario, the contributions of the bottom 20% of the population are about 9% of their income compared to 3.4% for the wealthiest 20% of the population (column g).

**Table 1.** Illustrating misconceptions about equitable health financing using a hypothetical example

	(a) Share of total population (%)	(b) Share of total income (%)	(c) Average income per head ($)	(d) Average healthcare contributions (taxes) per head ($)	(e) Share of total healthcare contributions (taxes) (%)	(f) Average monetized healthcare benefits per head ($)	(g) Share of income spent on healthcare (taxes) (%)
Poorest 20%	20.00%	0.36%	1063.36	94.12	0.72%	145.60	8.87%
Next poorest 20%	20.00%	1.98%	5888.43	490.70	3.73%	780.34	8.34%
Middle 20%	20.00%	6.52%	19 449.42	1503.62	11.43%	1600.24	7.76%
Q4	20.00%	17.21%	51 290.84	3613.63	27.46%	1754.21	7.06%
Richest 20%	20.00%	73.94%	220 395.09	7456.38	56.67%	2101.23	3.40%
Total	100.00%	100.00%	59 617.43	2631.69	100.00%	1276.32	4.42%

*Source*: Authors’ compilation using simulated hypothetic data.

### Misconception 2 (pro-poor)

It is always equitable and fair when the share of total healthcare contributions by poorer population groups is lesser than the share contributed by more affluent population groups.

This misconception, termed the pro-poor misconception, is illustrated in Table 1 (column e). Here, because the share that the bottom 20% of the population contributes towards health services is lesser than others, it may be misconstrued as ‘equitable’. In this case, the wealthiest 20% of the population accounts for over 56% of healthcare contributions compared to <1% attributed to the poorest 20%. Unfortunately, while this shows a ‘favourable’ gradient for the poor, it is not equitable. In this case, when column e is compared to column b (Table 1), it becomes clear that the share of income that the bottom 20% of the population has is lesser than their share of healthcare contributions. Also, the top 20% of the population has about 74% of total income but accounts for <60% of total contributions towards healthcare.

Using Ghana as a case study, as shown in Table 2, the share of out-of-pocket health spending by poorer groups is lesser than their population share (Akazili *et al.*, 2011). For example, the poorest 20% of Ghanaians account for 6.9% of out-of-pocket health spending compared to the top 20% accounting for 42%. It is misleading to conclude that out-of-pocket health spending is equitable or fair in Ghana because the gradient ‘favours’ poorer groups. Comparing the distribution of income and out-of-pocket health spending shows how inequitable out-of-pocket health spending is in Ghana (Akazili *et al.*, 2011). Moreover, poorer groups may face a relatively high burden of unmet needs and may not afford out-of-pocket costs for health services, hence their relatively small share.

**Table 2.** Out-of-pocket health spending and income distribution in Ghana

	Share of the total population (%)	Share of income (%)	Share of out-of-pocket health spending (%)
Poorest 20%	20%	5.5%	6.9%
Next poorest 20%	20%	10.1%	12.4%
Middle 20%	20%	14.7%	18.4%
Q4	20%	21.3%	20.2%
Richest 20%	20%	48.4%	42.1%

*Source*: Recomputed using Table 3 in Akazili *et al*. (2011).

### Misconception 3 (cross-subsidization)

It is always equitable and fair when the healthcare contributions per head by poorer population groups are lesser than the monetized healthcare benefit they received compared to more affluent groups, irrespective of how contributions to health services are structured.

To understand the misconception of cross-subsidization, consider when health service use is monetized for population groups like quintiles (e.g. as it is done for benefit incidence analysis where every health service utilization is assigned a monetary value; McIntyre and Ataguba, 2011). Further, this monetized health service use is compared with each population group’s contributions to financing health services. A misleading conclusion may arise that health financing is always fair when the monetary benefits that poorer population groups receive exceed the amount they contributed per head to finance health services. This can be seen by comparing columns f and d (Table 1). From a policy perspective, it may be desirable for poorer groups to benefit more than they contribute to the system, mainly because they bear a higher burden of disease than wealthier groups (Deaton, 2002). However, this scenario ignores the distribution of income and ill-health burden or need for healthcare. It is crucial to distinguish between the principle for assessing equity in health service utilization and that for equity in health financing, even though they may be interrelated. An individual’s health service utilization should be based on need, while health service finance should be related to ability-to-pay. As described in this misconception, having a favourable redistribution where the benefits derived by poorer groups exceed their monetary contributions to financing health services should not supplant an equitable health financing system.

### When is health financing equitable?

Knowing that equitable or fair health financing requires that contributions to the health system are related to individuals’ or households’ ability-to-pay (Wagstaff and van Doorslaer, 1993) may not become evident, as demonstrated in Table 1. There is no consensus on the extent to which differential health service payments by poorer and richer groups constitute ‘fair or equitable financing’ (Ataguba *et al.*, 2018). That notwithstanding, using the information in Table 1, assessing equity in health financing involves comparing columns b and e. Health financing is equitable when the share of total contributions to financing health services by poorer population groups is lesser than their income share. It is not sufficient to look at the distribution in column e (share of healthcare contribution) in isolation from the nature of income distribution in the country (columns b and c). This is a very critical aspect of assessing equitable health financing in a country. So, when analysts look at healthcare contributions in isolation from income distribution in a country, it is not feasible to make valid conclusions about fairness in health financing. Stated differently, equity in health financing requires that ‘the share of income’ contributed towards financing healthcare be smaller for poorer than wealthier population groups (Ataguba *et al.*, 2018; Wagstaff *et al.*, 1989). This is what Ataguba *et al*. (2018) describe as ‘structural progressivity’. In addition to using formal indices such as the Kakwani and Suits indices, a couple of studies have analysed equity in health financing using structural progressivity by looking at the percentage of income contributed by different quintiles towards financing health services (Akazili *et al.*, 2011; Mtei *et al.*, 2012; Ataguba and McIntyre, 2018). Using the information in column g, for instance, financing health services via taxes is not equitable in this hypothetical example.

## Conclusion

Assessing health financing equity is critical for monitoring progress towards UHC. Policymakers and practitioners need to understand what constitutes an equitably financed health system to show the progressive realization of UHC and inform appropriate and timely policy choices. Misconceptions of fair contributions, pro-poorness and cross-subsidization, discussed in this paper, may make it difficult to fully understand an equitably financed health system and probably policy options to improve equity in health financing. It is crucial to highlight that judgement about how fair a health financing system is cannot be made without recourse to income distribution in a country.

## Reflexivity statement

Health equity and financing researchers from Africa wrote this paper. There is gender balance in authorship, with one male and one female researcher. J.E.A. is based at an academic institution, while G.A.K. is at an international organization. The paper benefitted significantly from the perspectives of two different authors, their knowledge of health equity and financing and policy engagements in Africa.
